# Consequences of GMPPB deficiency for neuromuscular development and maintenance

**DOI:** 10.3389/fnmol.2024.1356326

**Published:** 2024-02-14

**Authors:** Mona K. Schurig, Obinna Umeh, Henriette Henze, M. Juliane Jung, Lennart Gresing, Véronique Blanchard, Julia von Maltzahn, Christian A. Hübner, Patricia Franzka

**Affiliations:** ^1^Institute of Human Genetics, Jena University Hospital, Friedrich Schiller University, Jena, Germany; ^2^Institute of Diagnostic Laboratory Medicine, Clinical Chemistry and Pathobiochemistry, Charité-Universitätsmedizin Berlin, corporate member of Freie Universität Berlin, Humboldt-Universität zu Berlin, and Berlin Institute of Health, Berlin, Germany; ^3^Leibniz Institute on Aging - Fritz Lipmann Institute, Jena, Germany; ^4^Department of Human Medicine, Medical School Berlin, Berlin, Germany; ^5^Stem Cell Biology of Aging, Faculty of Health Sciences, Brandenburg Technische Universität Cottbus-Senftenberg, Senftenberg, Germany; ^6^Center of Rare Diseases, Jena University Hospital, Friedrich Schiller University Jena, Jena, Germany

**Keywords:** glycosylation, mannosylation, GMPPB, skeletal muscle, nervous system, myogenesis, neurogenesis

## Abstract

Guanosine diphosphate-mannose pyrophosphorylase B (GMPPB) catalyzes the conversion of mannose-1-phosphate and GTP to GDP-mannose, which is required as a mannose donor for the biosynthesis of glycan structures necessary for proper cellular functions. Mutations in GMPPB have been associated with various neuromuscular disorders such as muscular dystrophy and myasthenic syndromes. Here, we report that GMPPB protein abundance increases during brain and skeletal muscle development, which is accompanied by an increase in overall protein mannosylation. To model the human disorder in mice, we generated heterozygous GMPPB KO mice using CIRSPR/Cas9. While we were able to obtain homozygous KO mice from heterozygous matings at the blastocyst stage, homozygous KO embryos were absent beyond embryonic day E8.5, suggesting that the homozygous loss of GMPPB results in early embryonic lethality. Since patients with GMPPB loss-of-function manifest with neuromuscular disorders, we investigated the role of GMPPB *in vitro*. Thereby, we found that the siRNA-mediated knockdown of *Gmppb* in either primary myoblasts or the myoblast cell line C2C12 impaired myoblast differentiation and resulted in myotube degeneration. siRNA-mediated knockdown of *Gmppb* also impaired the neuron-like differentiation of N2A cells. Taken together, our data highlight the essential role of GMPPB during development and differentiation, especially in myogenic and neuronal cell types.

## Introduction

Glycosylation is one of the most common post-translational modifications of proteins and lipids, which can have important consequences for protein stability and conformation. It plays a prominent role in cell-to-cell communication, cell matrix interaction, adhesion, protein targeting and folding, viral or bacterial infection, progression of cancer and aging (Varki et al., 2009; Breloy and Hanisch, 2018). Abnormal glycosylation of proteins can induce deleterious effects as observed in congenital disorders of glycosylation (CDGs), which often result in serious, sometimes fatal malfunctions of different organ systems such as brain and muscle (Péanne et al., 2018). A typical example are mutations in the gene encoding the enzyme GDP-mannose-pyrophosphorylase-B (GMPPB). GMPPB is crucial for the conversion of mannose-1-phosphate and guanosine triphosphate into GDP-mannose, which is required as a mannose donor for glycosylation (Ning and Elbein, 2000). Bi-allelic mutations in GMPPB are associated with variable disorders such as muscular dystrophy and other neurological symptoms including intellectual disability (Carss et al., 2013; Belaya et al., 2015; Liu et al., 2021). Additional symptoms such as cerebellar hypoplasia, seizures, or cardiac involvement are reported for some patients. The age at onset and disease progression varies from early infancy to adulthood (Belaya et al., 2015; Jensen et al., 2015; Rodriguez Cruz et al., 2016; Balcin et al., 2017; Luo et al., 2017; Sun et al., 2020; Chompoopong and Milone, 2023).

Previous morpholino-based knockdown studies in zebrafish suggested that GMPPB is required for the development of motor neurons and myofibers (Carss et al., 2013; Liu et al., 2021; Zheng et al., 2021). Here, we report that the KO of GMPPB in mice results in early embryonic lethality, suggesting that protein mannosylation is essential for embryonic development and that the loss of GMPPB to provide activated mannose cannot be compensated during early development. Being unable to study the consequences for neuronal and muscular differentiation *in vivo*, we studied the consequences of the knockdown of *Gmppb* on myoblasts or N2A cells. Both, differentiation and viability of the cells was severely compromised upon knockdown of *Gmppb* emphasizing the essential role of GMPPB.

## Methods

All animal experiments were approved by the Thüringer Landesamt für Lebensmittelsicherheit und Verbraucherschutz (TLV). Experiments were performed in a C57BL/6 background. Mice were housed in a 12-hour light/12-hour dark cycle and had access to mouse chow *ad libitum*.

### Cell culture

N2A (ATCC) cells were cultured in DMEM Glutamax (Sigma-Aldrich) supplemented with 10% [v/v] FBS (Gibco), 1% [v/v] penicillin/streptomycin (Gibco) at 37°C. For differentiation, cells were treated with differentiation medium [Neurobasal medium 1g/l glucose (Gibco) + 1X B-27 (Gibco) + 1X sodium pyruvate (Gibco) + 1X glutamine (Gibco) + 1% [v/v] penicillin/streptomycin (Gibco)].

Embryonic stem (ES) cells were cultured in DMEM with high glucose, no sodium pyruvate and 25 mM HEPES (Invitrogen) supplemented with 15% [v/v] FBS (PAA Gold), 1% [v/v] penicillin/streptomycin (Gibco), 1X NEAA (Invitrogen), 1X sodium pyruvate (Invitrogen), 1X glutamine (Invitrogen), 1X Nulceosidmix (Invitrogen), 2 × 2-mercaptoethanol (Invitrogen) and 1000 U/ml LIF (Merck) @37°C.

Primary myoblasts isolated from WT mice were cultured in growth medium [F10, 20% (v/v) FBS, 2% penicillin/streptomycin, 2.5 ng/mL basic fibroblast growth factor (bFGF); all from Gibco] at 37°C. For differentiation, primary myoblasts were incubated with differentiation medium [DMEM, Sigma-Aldrich; 5% (v/v) horse serum, Gibco; 2% penicillin/streptomycin, Gibco].

C2C12 cells (ATCC) were seeded in growth medium (DMEM, Sigma-Aldrich; 10% [v/v] FBS, Gibco; 2% [v/v] penicillin/streptomycin; Gibco) at 37°C. For differentiation, cells were incubated with differentiation medium (DMEM, Sigma-Aldrich; 2% horse serum, Gibco).

### Targeted inactivation of the murine *Gmppb* gene

To disrupt GMPPB in mice, we targeted exon 4 of the murine *Gmppb* gene in embryonic stem cells (ES) by CRISPR/Cas9 with two sgRNAs (sgRNA1: gttgaacgaagaaagggt, sgRNA2: gtgcccgatgaaactgcacca) resulting in a 54 bp deletion. For this, ES cells were injected with a pSpCas9-(BB)-2A-Puro (PX459) vector (62988, Addgene) that contained both Cas9 and sgRNAs. Success of ES cell transfection was verified by polymerase chain reaction (PCR) using gcaaactttagggccagcaaa as forward primer and gaggtggagggtaccttag as reverse primer as well as by Western Blot and Sanger sequencing. Selected ES cells were then injected into foster mice and resulting chimeric mice were subsequently mated with C57BL/6 mice until reaching the fourth generation.

### N2A cell differentiation experiments for microscopic analysis

N2A cells were seeded in a 12-well cell culture plate. The next day, cells were treated with differentiation medium and transfected with siRNA against either control (siScr) (Dharmacon) or *Gmppb* (si*Gmppb*) (Thermo Fischer) according to the manufacturer’s protocol using lipofectamine 2000 (Invitrogen). After 4 days of differentiation, cells were fixed with 4% paraformaldehyde (PFA) and imaged in phosphate-buffered saline (PBS). Images were taken with a Keyence microscope BZ-X800E in the brightfield modus. N2A cell differentiation was morphologically evaluated by measuring the length and numbers of dendrite-like protrusions of first, second and third order protrusions. All single cells from at least eight images per condition and experiment were traced manually.

For measuring total protrusion numbers per neuron-like cell, all traced/visible protrusions per cell were counted for all single cells in at least eight images per condition and experiment. From these cells, the mean was determined for each condition/experiment.

For measuring the total protrusion length per cell, the length of all traced/visible projections per cell was measured for all single cells from at least eight images per condition and experiment. From these cells, the mean was quantified for each condition/experiment.

For assessing the mean protrusion length per order, the length of all traced/visible protrusions per respective protrusion order was measured for each cell. Per projection order, the mean length was measured from all assessed cells for each condition/experiment.

### N2A cell differentiation experiments for immunoblot analysis

N2A cells were seeded in 10 cm cell culture dishes (Greiner). The next day, cells were treated with differentiation medium and transfected with siRNA against either control (siScr) (Dharmacon) or *Gmppb* (si*Gmppb*) (Thermo Fischer) according to the manufacturer’s protocol using lipofectamine 2000 (Invitrogen). After 3 days of differentiation, cells were harvested and lysed in RIPA buffer [50 mM Tris-HCl pH 7.4, 150 mM NaCl, 1% (v/v) NP-40, 1% (w/v) sodium deoxycholate, 0.1% (w/v) SDS, 1 mM EDTA] and complete protease inhibitor (Roche). Cell homogenates were centrifuged at 10,000 g and the protein concentration in the supernatant was determined using the BCA assay kit (Thermo Fischer). Samples were stored at −20°C until further use.

### N2A neurite growth experiments for microscopic analysis

N2A cells were seeded in a 12-well cell culture plate. The next day, cells were treated with differentiation medium and allowed to differentiate for 3 days. Then, cells were transfected with siRNA against either control (siScr) (Dharmacon) or *Gmppb* (si*Gmppb*) (Thermo Fischer) according to the manufacturer’s protocol using lipofectamine 2000 (Invitrogen). After 4 days of differentiation, cells were fixed and processed as described above.

### Protein isolation from ES cells

ES cells were harvested and lysed in RIPA buffer [50 mM Tris-HCl pH 7.4, 150 mM NaCl, 1% (v/v) NP-40, 1% (w/v) sodium deoxycholate, 0.1% (w/v) SDS, 1 mM EDTA] and complete protease inhibitor (Roche). Cell homogenates were centrifuged at 10,000 g and the protein concentration in the supernatant was determined using the BCA assay kit (Thermo Fischer). Samples were stored at −20°C until further use.

### Protein isolation from brain

Pregnant mother mice or pups were sacrificed and brain as well as skeletal muscle tissue (*musculus quadriceps femoris*) from embryos or pups was isolated. Tissue lysates were prepared as described previously (Franzka et al., 2022). Shortly, samples were homogenized with the Potter S tissue homogenizer (Sartorius) in RIPA buffer [50 mM Tris-HCl pH 7.4, 150 mM NaCl, 1% (v/v) NP-40, 1% (w/v) sodium deoxycholate, 0.1% (w/v) SDS, 1 mM EDTA] and complete protease inhibitor (Roche). After sonication, homogenates were spun down at 16,900 g to remove nuclei and insoluble debris. Protein concentration in the supernatant was determined using the BCA assay kit (Thermo Fischer) and then stored at −80°C until further use.

### Western blot

Proteins were denatured at 90°C for 5 min in Laemmli buffer (4X Laemmli buffer: 50% glycerol, 5% SDS, 0.25% 1.5 M Tris pH 6.8, 30% β-mercaptoethanol, 0.001% bromophenol blue, ddH_2_O). After separation by SDS-PAGE, proteins were transferred onto PVDF membranes (Whatman). Membranes were blocked in 2% BSA and incubated with primary antibodies at appropriate dilutions overnight at 4°C. The following primary antibodies were used: rabbit anti-GMPPB (Proteintech) 1:500, rabbit anti-GAPDH (Proteintech) 1:1000, biotin Con A (Biozol) 1:300. Primary antibodies were detected with HRP-conjugated secondary antibodies or HRP-conjugated streptavidin. Detection was performed with the Clarity Western ECL Substrate Kit (BioRad). The quantification of bands was done with ImageJ.

### Immunofluorescence stainings of embryo sections

Pregnant mother mice were sacrificed and embryos at embryonal day 13.5 (E 13.5) isolated. Embryos were immediately frozen in Tissue Tek (Weckert Labortechnik) on dry ice and afterwards cryo-sectioned. Sections were dried, fixed in 4% PFA, permeabilized with 0.25% TritonX100, blocked in 5% normal goat serum (NGS) and stained over night at 4°C with primary antibodies or lectins followed by incubation with the corresponding secondary antibodies or streptavidin coupled to fluorophores (Invitrogen). Following primary antibodies/lectins were used: biotin Con A (Biozol) 1:100, rabbit anti-GMPPB (Proteintech) 1:100. Nuclei were stained with DAPI (10 μg/ml, Invitrogen). Images were taken with a Keyence microscope BZ-X800E.

### *In situ* hybridization

Pregnant mother mice were sacrificed and embryos at embryonal day 13.5 (E 13.5) isolated. Embryos were immediately frozen in liquid nitrogen and afterwards cryo-sectioned. Sections were dried, fixed in 4% PFA, permeabilized with 0.2 M HCL, blocked and hybridized with digoxigenin-labeled antisense and sense RNA probes. The riboprobes covered exon 1-6 of the *Gmppb* transcript.

### Myoblast and C2C12 differentiation experiments for microscopic analysis

All experiments were performed as described previously (Franzka et al., 2021). Primary myoblasts isolated from WT mice were seeded on collagen-coated culture dishes. Cells were treated with differentiation medium for 2 days. Then, cells were transfected with siRNAs against either control (siScr) (Dharmacon) or *Gmppb* (si*Gmppb*) (Thermo Fischer) according to the manufacturer’s protocol using lipofectamine 2000 (Invitrogen). After 4 days of differentiation, cells were fixed with 2% PFA, permeabilized, blocked, and stained with antibodies directed against myogenin (F5D, DSHB) 1:2 and myosin heavy chain (MF20, DSHB) 1:2 overnight at 4°C followed by an incubation with the corresponding secondary antibodies (Invitrogen). Nuclei were stained with DAPI (10°μg/mL, Invitrogen).

The fusion index was quantified as the number of nuclei inside myosin heavy chain (MYHC)-positive myotubes divided by the total number of nuclei per field of view.

The myotube diameter was assessed by measuring the maximal width of all MYHC-positive myotubes per field of view.

C2C12 cells were seeded and treated with differentiation medium and transfected with siRNAs against either control (siScr) (Dharmacon) or *Gmppb* (si*Gmppb*) (Thermo Fischer) according to the manufacturer’s protocol using lipofectamine 2000 (Invitrogen). After 4 days of differentiation, cells were fixed and processed as described above.

### Myoblast differentiation experiments for immunoblot analysis

Primary myoblasts isolated from WT mice were seeded and treated with differentiation medium for 2 days. Then, cells were transfected with siRNAs against either control (siScr) (Dharmacon) or *Gmppb* (si*Gmppb*) (Thermo Fischer) according to the manufacturer’s protocol using lipofectamine 2000 (Invitrogen). After 4 days of differentiation, cells were harvested and lysed in RIPA buffer [50 mM Tris-HCl pH 7.4, 150 mM NaCl, 1% (v/v) NP-40, 1% (w/v) sodium deoxycholate, 0.1% (w/v) SDS, 1 mM EDTA] and complete protease inhibitor (Roche). Cell homogenates were centrifuged at 10,000 g and the protein concentration in the supernatant was determined using the BCA assay kit (Thermo Fischer). Samples were stored at −20°C until further use.

### C2C12 maintenance experiments for microscopic analysis

C2C12 cells were seeded, treated with differentiation medium for 3 days and then transfected with siRNAs against either control (siScr) (Dharmacon) or *Gmppb* (si*Gmppb*) (Thermo Fischer) according to the manufacturer’s protocol using lipofectamine 2000 (Invitrogen). After 4 days of differentiation, cells were fixed and processed as described above.

### Blastocyst isolation and visualization

Heterozygous GMPPB KO mice were mated in a timed manner and blastocysts were flushed from the uteri of pregnant females on embryonic day E3.5. Single blastocysts were transferred into a 96-well plate using a mouth pipette. Blastocysts where then imaged with a Keyence BZ-X800E microscope.

After imaging, blastocysts were transferred into reaction vessels containing 5 ml of blastocyst lysis buffer [10 mM Tris pH 8.3, 50 mM KCl, 2.5 mM MgCl_2_, 0.45% NP-40, 0.45% Tween 20 and 0.2 mg/ml proteinase K in ddH_2_O] and lysed for 3 h at 55°C followed by inactivation at 85°C for 15 min. Blastocyst were genotyped via nested PCR. Following primer pairs were used: gagggatggatactgactg as forward primer and gaggtggagggtaccttag as revers primer for the first PCR as well as gaggtggagggtaccttag as forward primer and gcaaactttagggccagctc as revers primer for the second PCR.

### Statistical analysis

For statistical analysis, raw data were analyzed for normal distribution with the Kolmogorov–Smirnov test or by graphical analysis using the Box-Plot and QQ-Plot in Graphpad prism 9. If appropriate, we either used 1-way ANOVA, 2-way ANOVA, or two-tailed Student’s *t*-tests. * indicates *p* < 0.05, **p < 0.01, and ***p < 0.001. For statistical analysis, we used Graphpad prism 9. For all data, means with standard error of the mean (SEM) and individual data points with SEM are shown.

## Results

### Expression of GMPPB increases during murine brain and muscle development

**FIGURE 1:**
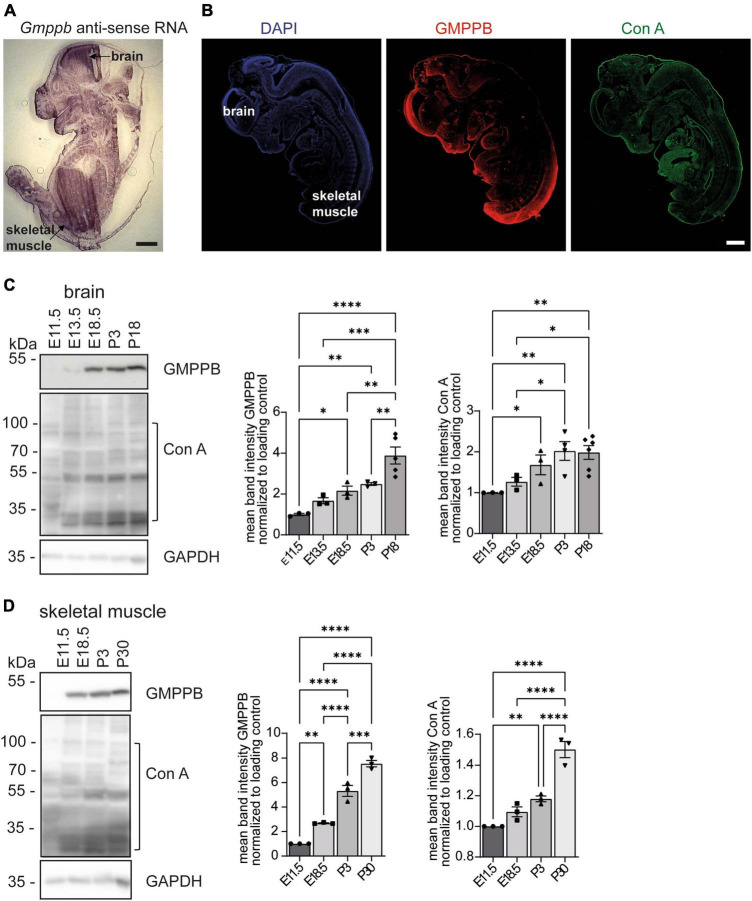
Expression of GMPPB increases during murine brain and skeletal muscle development. **(A)** *In situ* hybridization of an E13.5 murine embryo section with a *Gmppb*-specific antisense probe (scale bar: 500 μm). **(B)** Immunofluorescence labeling of nuclei (DAPI), GMPPB (red) and mannose residues in glycan structures (Con A, green). Scale bar: 500 μm. **(C)** Immunoblot analysis of brain tissues dissected at different time-points showing increasing signal intensities for GMPPB and Con A with increasing developmental stages. Black brackets indicate measured bands at indicated molecular weights. GAPDH served as loading control (*n* = 3–6 samples per developmental stage, 1-way-ANOVA with Fischer’s LSD test). **(D)** Immunoblot analysis of skeletal muscle (*musculus quadriceps femoris*) dissected at different time-points showing increasing signal intensities for GMPPB and Con A with further development. Black brackets indicate measured bands at indicated molecular weights. GAPDH served as loading control (*n* = 3 samples per developmental stage, 1-way-ANOVA with Fischer’s LSD test). Quantitative data are presented as mean ± SEM with individual data points. *P < 0.05; **P < 0.01; ***P < 0.001, ****P < 0.0001.

To analyze the expression pattern of GMPPB during embryonic mouse development, we performed *in situ* hybridizations of sagittal embryonic day 13.5 (E13.5) mouse sections with *Gmppb* specific probes (Figure 1A, Supplementary Figure 1A). Overall, the expression was very broad with a prominent labeling of the developing brain and skeletal muscles (Figure 1A, Supplementary Figure 1A). Staining of embryonic sections with an antibody directed against GMPPB confirmed a broad expression pattern (Figure 1B, Supplementary Figure 1B). The staining for GMPPB is in agreement with the Concanavalin A (Con A) staining, a lectin that specifically binds to mannose residues in glycan structures, broadly labelling E13.5 embryo sections (Figure 1B, Supplementary Figure 1B).

To study the expression of GMPPB at different stages of mouse development, we also assessed the abundance of GMPPB and the incorporation of mannose into glycan structures in embryonic and early postnatal (P) brain (Figure 1C) and skeletal muscle protein lysates (Figure 1D). GMPPB abundance strongly increased during embryonic as well as early postnatal development in both brain and skeletal muscles (Figures 1C, D). This upregulation was accompanied by an increase of mannose residues in glycan structures (Figures 1C, D). In summary, expression of GMPPB increases during murine brain and skeletal muscle development.

### Loss of GMPPB leads to embryonic lethality in mice

**FIGURE 2:**
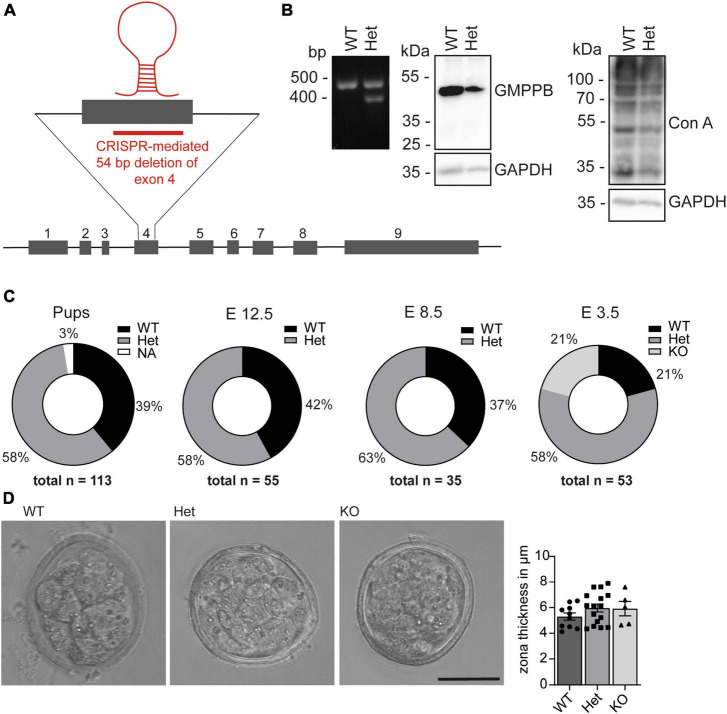
Loss of GMPPB leads to embryonic lethality in mice. **(A)** Genomic structure of the mouse *Gmppb* locus and the genome editing strategy. **(B)** Representative Ethidium bromide-stained agarose electrophoresis with the respective PCR products for GMPPB WT and Het ES cells. The immunoblot analysis suggests that the GMPPB protein abundance is reduced in Het compared to WT ES cells. In agreement, labeling for mannose residues in glycan structures was reduced. GAPDH served as loading control (*n* = 1). **(C)** Pie charts illustrating genotype percentages of WT, Het and KO GMPPB mice or embryos at different developmental stages [*n* (pups) = 113, *n* (E12.5) = 55, *n* (E8.5) = 35, *n* (E3.5) = 53]. **(D)** Representative images for WT, Het and KO blastocysts at E3.5 (scale bar: 50 μm) and quantification of the thickness of the zona pellucida (*n* = 5–16 blastocysts per genotype, 1-way-ANOVA with Fischer’s LSD test). Quantitative data are presented as mean ± SEM with individual data points.

To study the physiological role of GMPPB in mice we deleted 54 base pairs in exon 4 of *Gmppb* in mouse ES cells using CRISPR/Cas9, which inactivates the nucleotide transferase domain of GMPPB (Zheng et al., 2021; Figure 2A). The deletion was verified by PCR and Sanger sequencing (Figure 2B). Immunoblot analysis of protein lysates of wild-type (WT) and heterozygous (Het) ES cells using a polyclonal GMPPB antibody, which recognizes several epitopes in the protein, suggested a reduction in the abundance of GMPPB in Het cells (Figure 2B). Since no additional GMPPB-specific bands of lower size were detected in Het compared to WT cell lysates, it can be excluded that the deletion of 54 bp in exon 4 led to the expression of a variant truncated GMPPB protein. Concomitantly, mannosylation was reduced in Het cells (Figure 2B). Notably, we did not detect any homozygous deletions of the GMPPB allele in 384 analyzed individual ES cell clones (data not shown). Two independent heterozygous targeted ES cell clones were injected into blastocysts and transferred into foster mice. The resulting chimeric mice were subsequently mated with C57BL/6 mice for 3 generations. Heterozygous mice of the fourth generation were mated to obtain homozygous offspring. We did not detect any homozygous KO pups out of 113 genotyped newborn pups (Figure 2C). Analysis of embryos from terminated pregnancies from heterozygous matings at different embryonal time-points did not identify any homozygous KO embryos at E8.5 (35 genotyped embryos) or E12.5 (55 genotyped embryos) (Figure 2C). Only at E3.5, at the blastocyst stage, we were able to identify homozygous KO embryos. Overall, the structure and the size of blastocysts did not differ between genotypes (Figure 2D). Furthermore, the thickness of the zona pellucida, which is built up of glycoproteins, did not differ between the different genotypes (Figure 2D). Taken together, loss of GMPPB leads to early embryonic lethality in mice, but does not affect the size and shape of blastocysts.

### Knockdown of *Gmppb* affects N2A cell differentiation and growth

**FIGURE 3:**
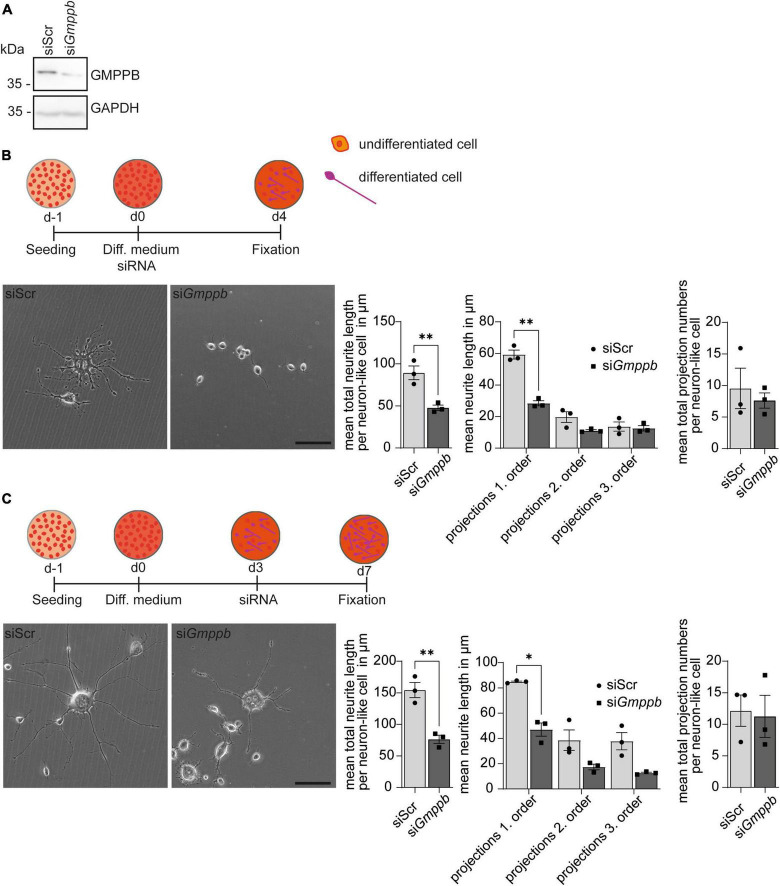
Knockdown of *Gmppb* affects N2A cell differentiation and neurite growth. **(A)** Immunoblot analysis confirming the knockdown of *Gmppb*. GAPDH served as loading control. **(B)** Experimental design, representative images of N2A cells (scale bar: 50 μm), and quantification of protrusion length and protrusion numbers (*n* = 3 experiments with 8–12 images per condition and experiment with 5–30 cells per image, Student’s *t*-test or 1-way ANOVA with Fischer’s LSD test). **(C)** Experimental design, representative images of N2A cells (scale bar: 50 μm), and quantification of protrusion lengths and numbers (*n* = 3 experiments with 8–12 images per condition and experiment with 1–15 cells per image, Student’s *t*-test or 1-way ANOVA with Fischer’s LSD test). Quantitative data are presented as mean ± SEM with individual data points. *P < 0.05; **P < 0.01.

To assess the role of GMPPB for neurite differentiation and growth *in vitro*, we transfected N2A cells with siRNA to knockdown *Gmppb* and followed their neuronal-like differentiation upon serum deprivation. Knockdown of *Gmppb* was confirmed by immunoblot analysis 4 days after transfection (Figure 3A).

To assess the role of GMPPB in neurite differentiation, we transfected N2A cells with siRNA directed to *Gmppb*, or a scrambled control at induction of differentiation (Figure 3B). After 4 days of differentiation, cells were fixed and analyzed. Neurite differentiation was morphologically evaluated by measuring the length and numbers of dendrite-like projections of first, second and third order. Therefore, all visible single cells in at least eight images per condition and experiment were manually traced. While control cells showed prominent dendrite-like structures after 4 days of differentiation, the projection length was significantly decreased upon knockdown of *Gmppb* (Figure 3B).

To assess the role of GMPPB for the protrusion growth of differentiated N2A cells, we first differentiated N2A cells for 4 days, before transfection with siRNA directed against *Gmppb* (si*Gmppb*) or a control siRNA (siScr) (Figure 3C). After four additional days, cells were fixed and analyzed. While control cells maintained long primary dendrite-like protrusions with dendritic-like arborization of higher orders, cells with a knockdown of *Gmppb* showed primary dendrite-like protrusions with a significantly reduced length of primary and higher order protrusions indicating a lower cell complexity (Figure 3C). In summary, knockdown of *Gmppb* compromises N2A cell differentiation and growth of neurite-like protrusions.

### Knockdown of *Gmppb* results in reduced myogenic differentiation and degeneration of myotubes

**FIGURE 4:**
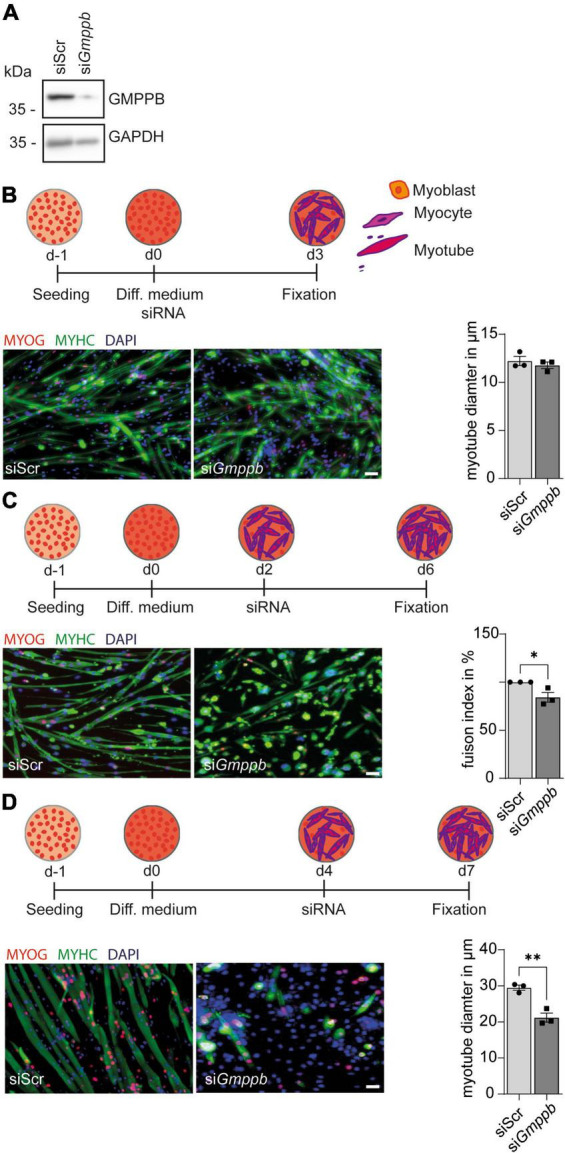
Knockdown of *Gmppb* results in reduced myogenic differentiation and degeneration of myotubes. **(A)** The knockdown of *Gmppb* in primary myoblasts was confirmed by immunoblot analysis. GAPDH served as loading control. **(B)** Experimental design and representative images of C2C12 cells stained for myogenin (MYOG) or myosin heavy chain (MYHC) (scale bar: 70 μm) and quantification of the myotube diameter (*n* = 3 experiments with 53–84 cells per condition and experiment, Student’s *t*-test). **(C)** Experimental design and representative images of primary mouse myoblasts stained for myogenin (MYOG) or myosin heavy chain (MYHC) (scale bar: 70 μm) and quantification of the fusion index (=nuclei in MHC-positive /all nuclei per field of view) (*n* = 3 experiments with 53–84 cells per condition and experiment, Student’s *t*-test). **(D)** Experimental design, representative images of C2C12 cells stained for myogenin (MYOG) or myosin heavy chain (MYHC) (scale bar: 70 μm), and quantification of the myotube diameter (*n* = 3 experiments with 53–84 cells per condition and experiment, Student’s *t*-test). Quantitative data are presented as mean ± SEM with individual data points. *P < 0.05; **P < 0.01.

Because patients with GMPPB variants do not only show neurological disorders, but also myopathic symptoms, we performed knockdown experiments in primary murine myoblasts or the myoblast cell line C2C12 followed by differentiation into myotubes. Also here, knockdown of *Gmppb* was efficient as verified by immunoblot analysis (Figure 4A). To assess if loss of GMPPB affects myogenesis *per se*, we investigated whether knockdown of *Gmppb* in myoblasts interferes with myogenic differentiation *in vitro*. Therefore, C2C12 cells were transfected with siRNA either targeting *Gmppb* or a scrambled control at the induction of myogenic differentiation (Figure 4B). Cells were stained for myogenin (MYOG), as marker for early myogenesis, namely myocytes, and myosin heavy chain (MYHC), a marker of terminally differentiated cells (myotubes). Notably, the myotube diameter was not affected by knockdown of *Gmppb* (Figure 4B) suggesting that induction of myogenic differentiation does not depend on GMPPB.

However, when mononucleated primary myocytes were transfected with a siRNA targeting *Gmppb*, we detected a decreased fusion index of myoblasts as a marker for myogenic differentiation (Figure 4C). This suggests that GMPPB is important for fusion of myoblasts with already existing myocytes or of myocytes with each other.

We next wondered whether differentiated myotubes are affected by loss of GMPPB as a measure of maintenance of myofibers *in vivo*. Therefore, we used C2C12 cells, a cell line derived from primary murine myoblasts, which are very similar to primary myoblasts but larger in size. To this end, C2C12 cells were differentiated into multinucleated myotubes, before they were transfected with a siRNA either targeting *Gmppb* or a scrambled control (Figure 4D). Thereby, we found that myotube diameter was significantly decreased after knock-down of *Gmppb* expression and that the number of myotubes was reduced in this condition (Figure 4D). These data suggest that the maintenance of differentiated myotubes depends on GMPPB. Taken together, loss of GMPPB affects fusion of myocytes and the size of late myotubes.

## Discussion

Disease associated GMPPB variants include missense, nonsense and frameshift mutations (Astrea et al., 2018; Tian et al., 2019) and are considered to result in GMPPB loss-of-function. Functional studies reported decreased enzymatic activities of approximately up to 90% (Liu et al., 2021). Here, we show that the total loss of GMPPB activity by disrupting the catalytic nucleotide transferase domain results in embryonic lethality in mice suggesting a pivotal role of GMPPB activity for early development. In agreement with this finding, no patients homozygous for a *GMPPB* KO allele have been reported up to now. Notably, loss of other enzymes important for mannosylation, such as phosphomannomutase 2 (PMM2) or phosphomannose isomerase (PMI), also result in embryonic lethality (DeRossi et al., 2006; Schneider et al., 2011; Sharma et al., 2014) further highlighting the essential role for mannosylation during early development.

While homozygous GMPPB KO embryos were still found at E3.5 (blastocyst stage), they were absent at E8.5. The fertilized egg (1-cell stage, zygote) starts dividing within a protective shell provided by the zona pellucida until the blastocyst stage (early blastocyst: 32-cell stage, late blastocyst: <100-cell stage) is reached and the embryo is released from the zona pellucida to implant into the uterine mucosa (Mihajlović and Bruce, 2017). Since the zona pellucida is mainly composed of glycoproteins (Bleil and Wassarman, 1980) and defects of the zona pellucida often result in early embryonic lethality (Shi et al., 2014; Wassarman and Litscher, 2022), we quantified the thickness of the zona pellucida of WT and KO blastocysts. Because we neither detected differences in the thickness of the zona pellucida nor in the cell numbers of E3.5 blastocysts (data not shown), the development up to the blastocyst stage appears to be grossly intact. So far, we were not able to exactly resolve when and why KO embryos are lost between E3.5 and E8.5. Possibly, the release of the embryo from the zona pellucida, i.e., the hatching, its implantation or its gastrulation is affected by GMPPB loss-of-function, which will require further analysis.

Since external mannose supplementation rescued embryonic lethality in PMM2-deficient mice (Schneider et al., 2011), it is tempting to speculate that mannose supplementation might be beneficial for GMPPB KO mice as well.

In agreement with a previous report for zebrafish (Liu et al., 2021), GMPPB expression in mice increases during embryonal and postnatal development, which is accompanied by an increase in mannosylated glycans. Since GMPPB loss-of-function in humans manifests with variable muscular and neurological defects (Belaya et al., 2015; Jensen et al., 2015; Rodriguez Cruz et al., 2016; Balcin et al., 2017; Luo et al., 2017; Sun et al., 2020; Chompoopong and Milone, 2023), we assessed the consequences of the knockdown of *Gmppb* in myoblasts or N2A cells. Indeed, knockdown of *Gmppb* in N2A cells and myoblasts affected both the development of dendrite-like protrusions in N2A cells as well as the differentiation of myotubes. Moreover, the maintenance of dendrite-like protrusions and the diameter of myotubes decreased upon knockdown of *Gmppb*. These findings might explain why the nervous system and skeletal muscles are affected in patients harboring GMPPB mutations. Several case studies reported patients with intellectual disability, cerebellar hypoplasia and/or cortical hypoplasia, epilepsy as well as gait abnormalities, muscle weakness, decreased skeletal muscle fiber diameter with hypoglycosylation of alpha-dystroglycan and reduced nerve conductance. In most cases, one or more of the mentioned central nervous system (CNS) disorders are accompanied by skeletal muscle abnormalities, but not necessarily vice versa (Carss et al., 2013; Raphael et al., 2014; Belaya et al., 2015; Cabrera-Serrano et al., 2015; Rodriguez Cruz et al., 2016). Of note, alpha-dystroglycan is expressed in both skeletal muscle and in the brain. It has been shown that decreased expression or altered glycosylation of alpha-dystroglycan affects myogenic differentiation (Chen et al., 2012) and compromises the assembly of neuromuscular junctions (Jacobson et al., 2001). In the brain, a lack or altered glycosylation of alpha-dystroglycan affects extracellular matrix components thereby altering cortical development (Michele et al., 2002; Moore et al., 2002).

Notably, symptom severity correlates with enzymatic activity of mutated GMPPB: mutations in the N-terminal nucleotidyl-transferase domain of GMPPB seem to impair its activity more severely as mutations in its C-terminal beta-helix domain (Liu et al., 2021). Hence, mutations in the catalytic domain of GMPPB normally result in CNS and muscle involvement, whereas mutations in the C-terminal part of GMPPB affect mostly only skeletal muscles (Sun et al., 2020). Interestingly, the most common mutations in GMPPB are c.79G >C (p.Asp27His) in the N-terminal part and c.860G > A (p.Arg287Gln) in the C-terminal part. More than 50% of reported patients are compound heterozygous for one of these two mutations (Chompoopong and Milone, 2023). Additional suggested hotspots include mutations in the N-terminal part of GMPPB [c.95C > T (Pro32Leu), c.308C > T (Pro103Leu), c.553C > T (arg185Cys)] (Sarkozy et al., 2018).

In summary, our study highlights the essential role of mannosylation during early stages of development. These findings not only foster our knowledge regarding the molecular mechanisms underlying GMPPB-related disorders but also provide a striking example how perturbations in post-translational modification can affect early development and cellular differentiation.

## Data availability statement

The raw data supporting the conclusions of this article will be made available by the authors, without undue reservation.

## Ethics statement

The animal study was reviewed and approved by the Thüringer Landesamt für Lebensmittelsicherheit und Verbraucherschutz (TLV). The study was conducted in accordance with the local legislation and institutional requirements.

## Author contributions

MS: Formal Analysis, Investigation, Writing – original draft. OU: Formal Analysis, Writing – original draft. HH: Investigation, Writing – original draft. MJ: Investigation, Writing – original draft. LG: Methodology, Writing – original draft. VB: Writing – original draft. JM: Investigation, Writing – original draft. CH: Conceptualization, Funding acquisition, Project administration, Supervision, Writing – original draft, Writing – review & editing. PF: Conceptualization, Formal Analysis, Funding acquisition, Investigation, Methodology, Project administration, Software, Supervision, Writing – original draft, Writing – review & editing.
