# Supplementary figures and images for: Consequences of GMPPB deficiency for neuromuscular development and maintenance
Source: Front Mol Neurosci. 2024 Feb 14;17:1356326. doi: 10.3389/fnmol.2024.1356326 (PMC10899408; doi:10.3389/fnmol.2024.1356326)

Figure 1C

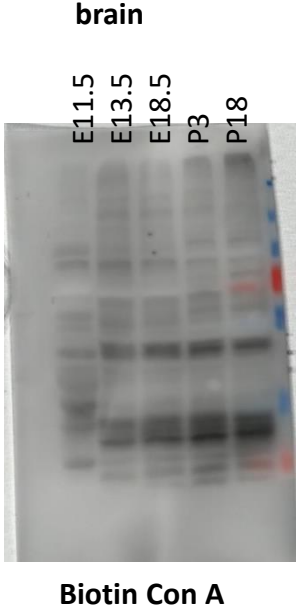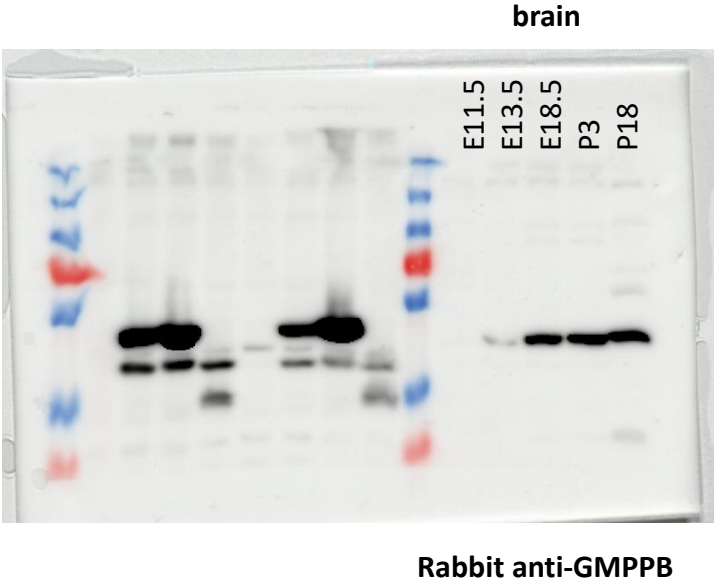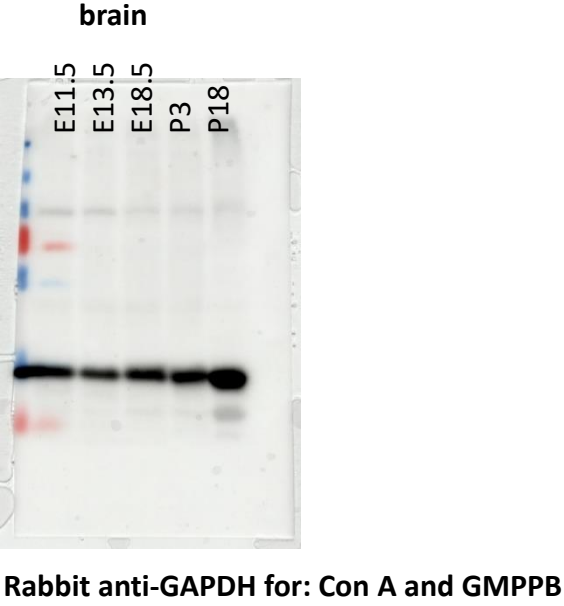

Figure 1D

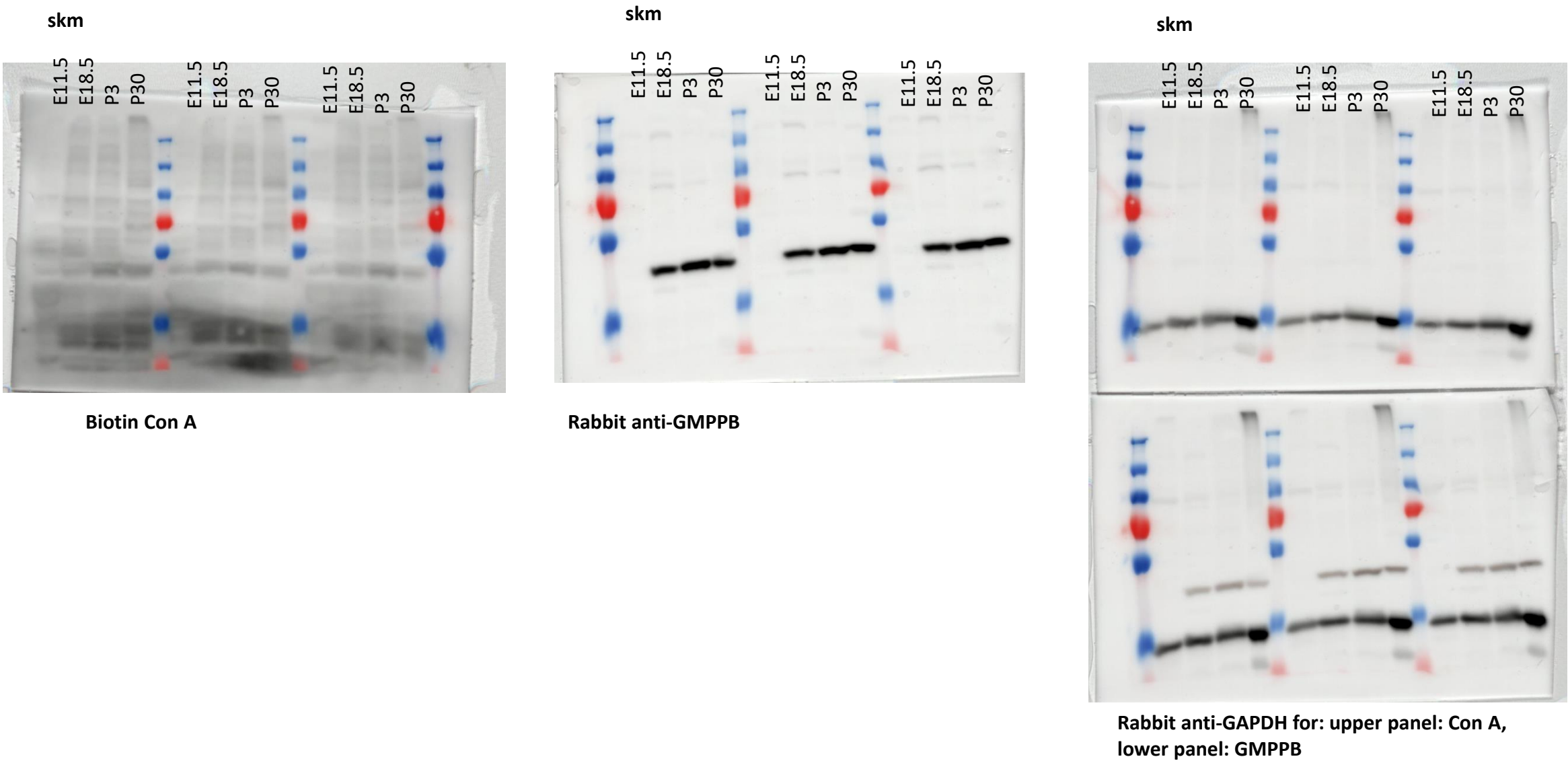

Figure 2B

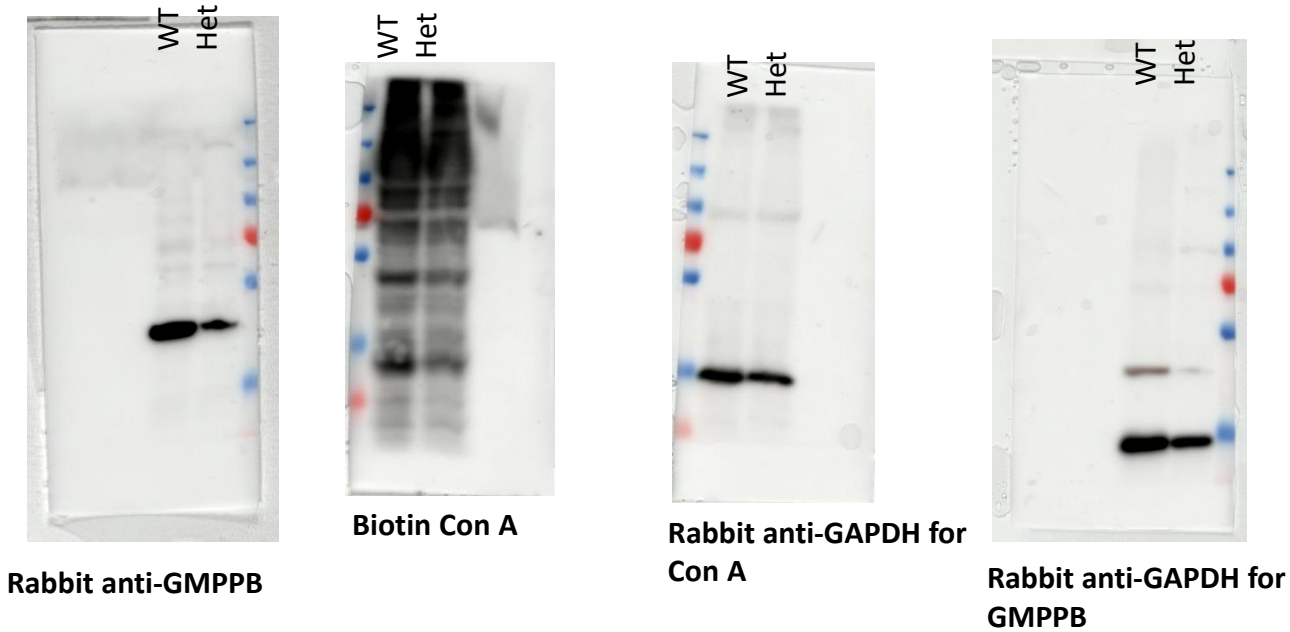

Figure 3A

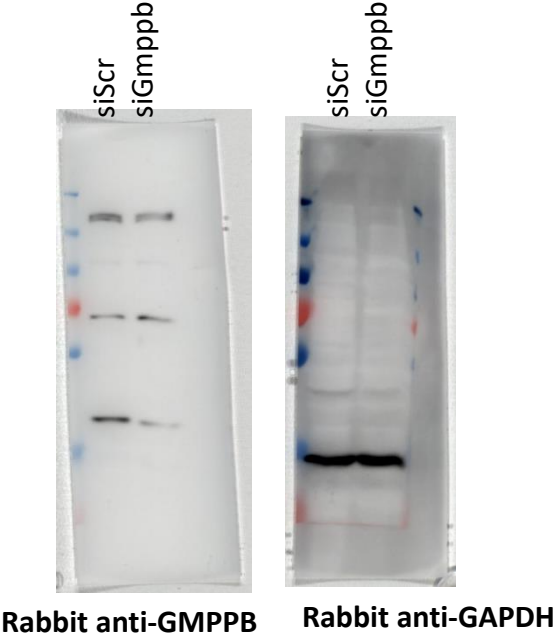

Figure 4A

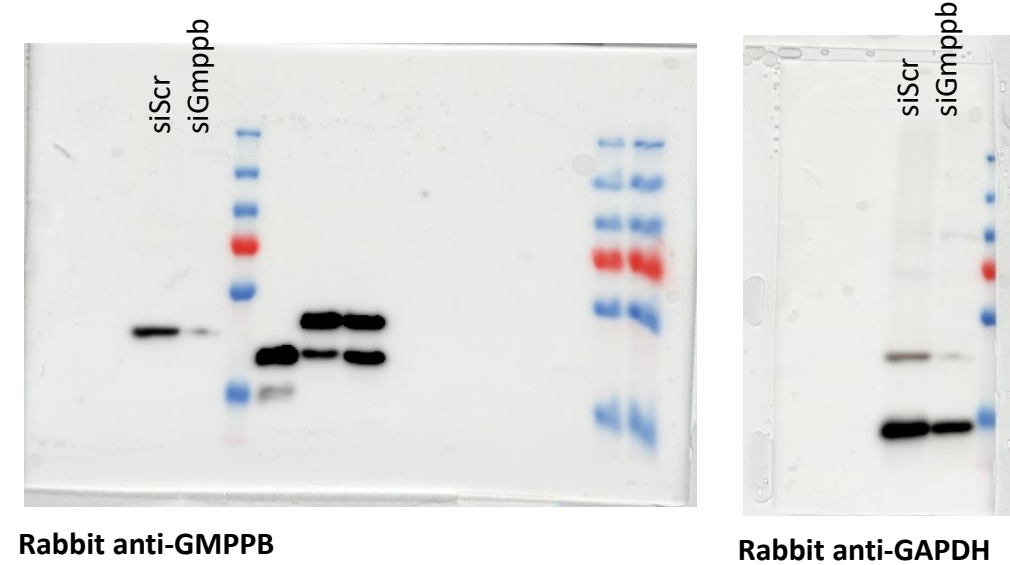

Supplement: Supplementary file 1 [file Data_Sheet_1.PDF]
