# Supplementary material for: Consequences of GMPPB deficiency for neuromuscular development and maintenance
Source: Front Mol Neurosci. 2024 Feb 14;17:1356326. doi: 10.3389/fnmol.2024.1356326 (PMC10899408; doi:10.3389/fnmol.2024.1356326)
Supplement: Supplementary file 2 [file Data_Sheet_2.PDF]

Supplementary Material

Consequences of GMPPB deficiency for neuromuscular development and maintenance

Mona K. Schurig[1], Obinna Umeh[2], Henriette Henze[3#], M. Juliane Jung[3#], Lennart Gresing[1], Véronique Blanchard[2, 4], Julia von Maltzahn[3, 5], Christian A. Hübner[1#], Patricia Franzka[1#*]

1 Institute of Human Genetics, University Hospital Jena, Friedrich Schiller University, Jena, Germany.
2 Charité-Universitätsmedizin Berlin, corporate member of Freie Universität Berlin, Humboldt-Universität zu Berlin, and Berlin Institute of Health, Institute of Diagnostic Laboratory Medicine, Clinical Chemistry and Pathobiochemistry, Berlin, Germany.
3 Leibniz-Institute on Aging — Fritz-Lipmann-Institute, Jena, Germany.
4 Medical School Berlin, Department of Human Medicine, Berlin, Germany
5 Stem Cell Biology of Aging, Faculty of Health Sciences, Brandenburg Technische Universität Cottbus-Senftenberg, Germany

# authors contributed equally
*** Correspondence:**
Patricia Franzka
patricia.franzka@med.uni-jena.de

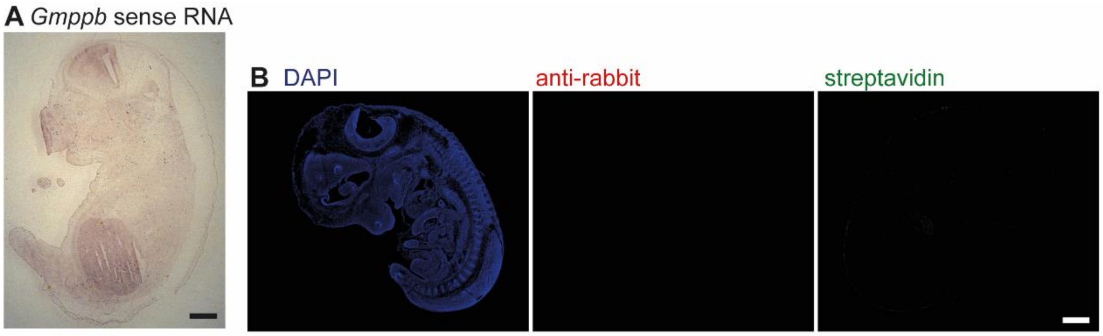

Supplementary Figure 1. GMPPB expression increases during murine brain and muscle development. A) *In situ* hybridization of an E13.5 murine embryo section with a *Gmppb*-specific sense probe (scale bar: 500 µm). **B)** Immunofluorescence stainings for nuclei (DAPI), anti-rabbit-coupled 555 Alexa Fluorophore and streptavidin-coupled 488 Alexa Fluorophore (scale bar: 500 µm).

Supplementary Figure 2. Full Western Blots.
